# Social Representations of Bedside Milk Expression Among Mothers of Preterm Newborns in Neonatal Intensive Care Units

**DOI:** 10.1111/jan.70228

**Published:** 2025-09-16

**Authors:** Ana Karen de Sousa Alves, Ana Paula Melo Façanha, Elaine Meireles Castro, Keline Soraya Santana Nobre, Flávia Vasconcelos Teixeira, Maria Milena Farias de Souza Castro, José Mateus Pires, Victórya Suéllen Maciel Abreu, Priscila de Souza Aquino

**Affiliations:** ^1^ Assis Chateaubriand Teaching Maternity Hospital Fortaleza Ceará Brazil; ^2^ Nursing Departament Federal University of Ceará Fortaleza Ceará Brazil

**Keywords:** bedside milk expression, breastfeeding, maternal behaviour, neonatal intensive care units, nursing

## Abstract

**Aim:**

To understand the social representations of bedside milk expression (BME) among mothers of preterm newborns in neonatal intensive care units (NICUs).

**Design:**

Qualitative descriptive study.

**Methods:**

The study was conducted from July to August 2024 in two NICUs of a referral maternity hospital in Fortaleza, Brazil. Nineteen mothers of hospitalised premature newborns participated. Semi‐structured interviews were conducted and subjected to thematic content analysis.

**Results:**

Mothers perceived BME as a meaningful act of protection and bonding, though some were unfamiliar with the practice. Emotional ambivalence was common, shaped by prior breastfeeding experiences and the context of prematurity. Discomfort related to privacy and shared spaces was noted. Support from healthcare professionals was essential to promote understanding and adherence.

**Conclusion:**

Social representations of BME are shaped by emotional, social and institutional experiences. Anchored in prior breastfeeding experiences and cultural meanings of maternal care, the practice is objectified through both gestures of affection and tangible barriers.

**Implications for the Profession and/or Patient Care:**

Healthcare professionals, particularly nurses, should receive training to support mothers in BME. Structural improvements, privacy and emotional support are essential for fostering maternal autonomy and confidence.

**Impact:**

This study highlights the barriers to BME, emphasising the role of healthcare support and the need for better infrastructure, privacy and training to enhance maternal confidence and breastfeeding.

**Reporting Method:**

The study followed the Consolidated Criteria for Reporting Qualitative Research checklist.

**Patient or Public Contribution:**

None.

**What Does This Paper Contribute to the Wider Global Clinical Community?:**

This paper highlights the pivotal role of healthcare professional support in overcoming barriers to BME and promoting breastfeeding practices.

**What Already Is Known?:**

Fresh breast milk is considered the gold standard for reducing complications and improving survival in preterm infants. BME is recommended as an effective strategy to ensure the availability of fresh breast milk. Mothers' social representations of this practice remain underexplored within the neonatal intensive care context.

**What This Paper Adds?:**

Explores mothers' social representations of BME in NICUs, addressing a significant gap in qualitative research. Reveals how emotional, social and institutional factors shape mothers' perceptions, motivations and challenges related to BME. Highlights the need for targeted professional support, improved infrastructure and privacy to enhance maternal autonomy and adherence to milk expression practices.

**Implications for Practice:**

Healthcare professionals, particularly nurses, should receive specialised training to provide technical guidance and emotional support, enhancing mothers' confidence and autonomy in BME. Improving infrastructure and ensuring privacy in NICUs are crucial to creating supportive environments that facilitate milk expression and strengthen maternal–infant bonding. Institutional policies should integrate maternal‐centred strategies to support breastfeeding continuity and promote humanised neonatal care.

**Summary**

- Provides an in‐depth understanding of how mothers of preterm infants socially construct the meaning of bedside milk expression, revealing symbolic, emotional and cultural aspects that influence adherence and inform the humanisation of NICU care.
- Identifies structural and interpersonal barriers while demonstrating the pivotal role of nursing teams in supporting and influencing maternal perceptions, strengthening breastfeeding self‐efficacy and promoting fresh breast milk provision to improve neonatal outcomes.

## 1 Introduction

Breast milk plays a crucial role in reducing complications associated with prematurity, including necrotising enterocolitis, late‐onset sepsis and retinopathy of prematurity (WHO and UNICEF 2020). Many neonates admitted to neonatal intensive care units (NICUs) are unable to feed orally during certain stages of hospitalisation due to physiological immaturity, clinical instability or underlying medical conditions. In these situations, enteral feeding via orogastric or nasogastric tubes is employed to ensure adequate nutritional support (Briere and Gomez 2024).

When fresh maternal milk is unavailable, pasteurised donor milk is the recommended alternative; however, it is not equivalent. Fresh maternal milk remains the gold standard for preterm infants due to its superior immunological and nutritional properties (Vargas 2022). Evidence shows that prolonged freezing and Holder pasteurisation degrade critical bioactive components, including antioxidant properties, which are essential for vulnerable preterm neonates (García‐Lara et al. 2012; Lev et al. 2014; Juncker et al. 2021). A cohort study involving 157 infants born before 32 weeks of gestation and weighing less than 1500 g found that those fed fresh maternal milk had a 2.6‐fold higher complication‐free survival rate compared to those receiving pasteurised milk, even after adjusting for confounding factors (Huang et al. 2023).

These findings reinforce the importance of fresh maternal milk and underscore the relevance of bedside milk expression (BME) which is the act of expressing milk at the infant's bedside. Endorsed by the Global Network of Human Milk Banks, BME facilitates the timely availability of fresh milk for preterm infants. Although BME can be practised in various hospital settings, its implementation in NICUs remains particularly significant due to the fragility and needs of preterm neonates (Brazil 2017).

However, providing breast milk in the context of NICU hospitalisation is complex. Emotional distress related to maternal–infant separation, such as anxiety, stress and depressive symptoms, can hinder maternal involvement and breastfeeding initiation (Yang et al. 2019). Despite being recognised as a best practice, BME may be compromised by structural limitations and insufficient training of healthcare teams. While prior studies have addressed the emotional and logistical challenges of milk expression (Brødsgaard et al. 2022; Li et al. 2024), little attention has been paid to the symbolic and social meanings embedded in the practice within the NICU context.

BME in NICUs extends beyond a technical practice; it carries symbolic, emotional and social meanings. The Social Representations Theory (SRT), developed by Moscovici (2007), provides a theoretical lens through which to understand how individuals and groups make sense of unfamiliar or abstract phenomena via the processes of anchoring, which consists of assimilating new experiences into pre‐existing knowledge structures, and objectification, which would be the materialisation of ideas into concrete images and practices. In this study, SRT facilitates the interpretation of how mothers of preterm infants construct meaning around BME, navigating between societal expectations, institutional environments and personal emotions.

When faced with the hospitalisation of a preterm newborn, a mother's feeding decisions are shaped not only by clinical recommendations but also by her prior knowledge and the sociocultural context in which she is embedded (Ikonen et al. 2015). Understanding the social representations that influence maternal perceptions of BME can inform more nuanced, respectful and effective care strategies.

This study seeks to expand current knowledge by understanding the social representations of BME among mothers of preterm infants hospitalised in NICUs. By uncovering how these meanings are constructed and shared, this research aims to enhance support strategies, strengthen maternal guidance and improve adherence to milk expression practices. Moreover, it contributes to a priority area aligned with Sustainable Development Goal 3, which emphasises reducing neonatal mortality through improved care and early‐life interventions.

In addition, Li et al. (2024) conducted a systematic review of qualitative studies on mothers' experiences with breast milk extraction during separation from their hospitalised babies and found no studies conducted in Brazil, which highlights the gap in the local context.

Given this context, this study aims to understand the social representations of BME among mothers of preterm newborns in NICUs.

## 2 Methods

### 2.1 Study Design

This study was conducted through a qualitative descriptive analysis based on the SRT. SRT was chosen as the theoretical framework because it focuses on identifying collective knowledge and thoughts that impact the implementation of BME. The study was developed according to the Consolidated Criteria for Reporting Qualitative Studies (COREQ) (Souza et al. 2021).

### 2.2 Setting and Participants

Data collection was conducted on‐site from July to August 2024 in two NICUs of a reference maternity hospital in Fortaleza, Ceará, Brazil. The hospital has a neonatal care department composed of five units (two Neonatal Intensive Care Units—NICUs, two Intermediate Care Units—UCINCOs and one Kangaroo Intermediate Care Unit—UCINCA). As a referral maternity hospital for high‐risk pregnancies, it frequently admits newborns with prematurity and congenital malformations. The NICUs have a total of 21 beds, with an average active bed occupancy rate of 114.52% during July and August 2024, the data collection period (Ebserh 2024).

To ensure participant anonymity, flower names were assigned to refer to them throughout the study. The study population consisted of mothers of preterm newborns admitted to NICUs who met the inclusion and exclusion criteria during the data collection period. The sample was selected by convenience, using a non‐probabilistic approach based on theoretical saturation.

The inclusion criteria were: being over 18 years old; being able to breastfeed according to the criteria established by the Brazilian Ministry of Health (Brazil 2015); being the mother of a preterm newborn with a gestational age between 25 weeks and 36 weeks and 6 days, as this period presents significant survival rates (BSP 2021); and being within 28 days postpartum at the time of the interview, as this is the period in which the baby is still considered a newborn (Hockenberry et al. 2023).

Mothers with any disabilities or health conditions preventing them from attending NICUs for BME, as well as those whose newborns had clinical conditions preventing them from receiving expressed human milk at the bedside, were excluded from the study. The final sample consisted of 19 participants.

### 2.3 Data Collection Procedure

Data collection was conducted by a nurse specialised in Neonatology, who had completed training in qualitative research methods. A sociodemographic and obstetric characterisation form, adapted from Splinter et al. (2018) and Paula (2022), was employed, along with a semi‐structured interview guide adapted from Garcia Junior (2015) and Paula (2022). The interview guide included topics related to participants' understanding of BME and their feelings about the practice. A pilot test was conducted with one mother to assess the instruments' comprehension, application time and the need for potential adjustments.

Participants were actively recruited within the hospital. Those who met the inclusion and exclusion criteria were invited to participate. Upon agreeing, the study objectives, risks and benefits were explained, and the Free and Informed Consent Form was signed in duplicate, with one copy provided to the participant. After completing the sociodemographic and obstetric characterisation form, the semi‐structured interviews were conducted in private spaces available within the neonatal units to ensure confidentiality and participant comfort. Participants' statements were recorded using a mobile device voice recorder and transcribed verbatim on the same day they were collected, which ensures fidelity to the participants' words and reduces interpretation bias during qualitative analysis.

### 2.4 Data Analysis

To describe participant characteristics, a descriptive statistical analysis of sociodemographic and obstetric data was performed using REDCap, including absolute and relative frequencies, as well as measures of central tendency. Qualitative data from the semi‐structured interviews were processed in IRaMuTeQ software (version 0.7 alpha 2), using descending hierarchical classification (DHC). This technique organises text segments into groups according to the similarity of the vocabulary used, allowing for the identification of discourse patterns shared among participants. This form of data analysis ensures an objective classification of text segments based on word frequency and co‐occurrence, also serving as a means to mitigate potential interpretation bias in the data (Sousa 2021).

DHC was chosen for its ability to structure large volumes of textual data into meaningful classes in an inductive and data‐driven way. The software highlights the most representative words of each class, which, alongside the corresponding text segments, support the interpretation of meaning (Sousa 2021). Words with a chi‐square (*χ* ^2^) ≥ 3.84 and *p* < 0.05 were considered statistically significant for class description (Góes et al. 2021).

Based on this output, the researchers named and categorised each class by examining the most characteristic terms and reading the associated excerpts, in line with thematic‐categorical content analysis as proposed by Bardin (2016). According to the author, content analysis involves three systematic phases: (1) pre‐analysis, which included a comprehensive reading of the transcribed interviews, organisation of the corpus and definition of analytical units; (2) exploration of the material, in which text segments identified by IRaMuTeQ were carefully examined and coded, grouping them into classes based on semantic similarity and (3) treatment of results and interpretation, where the researchers synthesised the meanings emerging from each class and organised them into thematic categories (Bardin 2016).

This iterative process was guided by the SRT, which served as both the theoretical and methodological framework, ensuring methodological rigour and analytical consistency. It was conducted by the same nurse specialist, who is the principal researcher, together with a nurse holding a PhD and experienced in qualitative research using IRaMuTeQ software.

### 2.5 Rigour and Trustworthiness

To ensure methodological rigour, we followed the four trustworthiness criteria proposed by Lincoln and Guba (1985). Credibility was strengthened through the verbatim transcription of interviews, lexical processing using IRaMuTeQ with DHC and thematic interpretation guided by SRT. The principal researcher, a neonatal nurse specialist, conducted all interviews and spent extended time in the field, which facilitated rapport and trust with the participants. Additionally, peer debriefing was carried out in collaboration with a nurse researcher with a PhD and extensive experience in qualitative studies to validate the thematic categories and interpretations.

Transferability was supported by providing a detailed description of the study setting, participant characteristics and data collection procedures, enabling readers to assess the applicability of the findings to similar clinical contexts. Dependability was ensured through systematic documentation of all methodological decisions and the maintenance of an audit trail, including interview transcripts, IRaMuTeQ outputs and dendrograms. Finally, confirmability was achieved through collaborative analysis between researchers and reflective discussions to minimise potential biases, with interpretations grounded in verbatim excerpts from participants' narratives, ensuring transparency and neutrality of the findings.

### 2.6 Ethical Considerations

The Research Ethics Committee of the Assis Chateaubriand Teaching Maternity Hospital, Fortaleza, Ceará, Brazil approved this study (approval number 6.918.137, 28 June 2024). The study complied with the bioethical principles established by Resolution No. 466/12 of the National Health Council, Brazil. All participants signed the Free and Informed Consent Form prior to data collection and confidentiality and anonymity were ensured by assigning flower names to participants.

## 3 Results

Among the 19 study participants, there was a predominance of married women (*n* = 8; 42%), with incomplete elementary education (*n* = 9; 47%), residing in the state capital (*n* = 16; 84%) and a median age of 32 years. Most births were caesarean sections (*n* = 14; 74%), with a gestational age (GA) ≥ 32 weeks in more than half of the cases (*n* = 10; 53%). Additionally, 10 participants (52.6%) had never performed BME. The newborns had a median age of 7 days at the time of data collection.

The result generated by DHC showed a retention rate of 77.13%, which is considered adequate according to Sousa (2021). Through DHC analysis, a manually constructed dendrogram was created (Table 1) to facilitate the interpretation of the results generated by IRAMuTeQ software. The software identified six classes, represented by the most significant words that demonstrated associations with one another.

**TABLE 1:** Dendrogram of word classes obtained through DHC.

Social representations of bedside milk expression (BME) in neonatal intensive care units											
Perceptions, beliefs and values regarding the practice of bedside milk expression (BME)				Incentives, barriers and perceived improvements for the implementation of bedside milk expression (BME)						The influence of the healthcare team on the implementation of bedside milk expression	
Class 1 (15.17%)		Class 4 (23.45%)		Class 5 (12.41%)		Class 3 (12.41%)		Class 2 (24.83%)		Class 6 (11.72%)	
Definition of BME		Perceptions and beliefs		Importance of BME		Suggested improvements for the implementation of BME		Incentives for the implementation of BME		Healthcare team	
Words	*χ* ^2^	Words	*χ* ^2^	Words	*χ* ^2^	Words	*χ* ^2^	Words	*χ* ^2^	Words	*χ* ^2^
Human milk bank	30.64	Time	27.67	Breastfeeding	25.98	Mother	45.09	Important	35.75	Experience	31.01
Stimulate	23.00	Dieta zero	13.43	Essential	21.76	Improved	28.95	Encourage	27.96	Calm	30.97
Breast milk	18.88	Hour	13.43	Nutrient	21.76	Speak	26.04	Better	17.52	Start	25.33
Process	17.13	Express milk	12.50	Look	19.54	Child	21.76	Month	15.68	Team	23.07
Think	16.91	Take	10.78	Understand	19.54	Want	14.90	Think	14.55	Scary	23.07
Come out	14.74	Manage	10.19	Love	16.94	Place	17.82	Baby	12.81	Reception	23.07
NICU	13.35	Let down	10.00	Good	9.05	Breastfeed	13.53	Importance	12.27	Super	15.91
Speak	13.35	Held	10.00	Prevent	8.29	Need	8.29	Bedside milk expression	9.67	Willingness	15.91
Express	12.89	Us	9.11	Develop	5.34	Great	8.29	Influence	9.28	Shame	14.66
Hand	11.44	Breast milk	6.67			Location	8.29	Health	8.56	Doctor	14.66
Nurse	10.07	Happy	6.51			Give up	8.29	Improvement	8.45	Information	8.86
Know	6.65	Stay	6.11			Contact	4.90	Bond	7.87	Nursing team	8.62
Milk expression	6.31	Start	6.09			Relationship	4.90	Incentive	5.87	Pass	6.98
Extraction	6.31	Hold	4.65					Formula	5.55	Wonderful	5.82
Nursing team	5.08	Interest	3.85					Come	4.85	Feel	5.00
								Breast	4.44	Leave	4.00
								Moment	4.12	Maternity	4.00
										Schedule	4.00
										Us	3.95

Abbreviations: *χ* ^2^, chi‐square; DHC, descending hierarchical classification; NICU, neonatal intensive care unit.

To name the classes, a thorough and exhaustive reading of the main associated words and the contextual discourse in which they were embedded was conducted to extract their semantic meaning (Góes et al. 2021). Thus, the classes were named as follows: Class 1—Definition of bedside milk expression; Class 2—Incentives for performing bedside milk expression; Class 3—Suggested improvements for performing bedside milk expression; Class 4—Perceptions and beliefs; Class 5—Importance of bedside milk expression; Class 6—Healthcare team.

Subsequently, based on the thematic‐categorical content analysis, the classes were grouped into three thematic categories to facilitate the discussion of the results: ‘Perceptions, beliefs and values regarding the practice of bedside milk expression’ (Classes 1 and 4); ‘Incentives, barriers and perceived improvements for the implementation of bedside milk expression’ (Classes 5, 2 and 3); and ‘The influence of the healthcare team on the implementation of bedside milk expression’ (Class 6). These categories were interpreted in light of the core constructs of STR.

Additionally, a visual representation of the findings was developed, integrating the three thematic categories and their interrelations in light of SRT, illustrating how the central and peripheral elements identified in the mothers' narratives converge to construct shared meanings regarding BME.

### 3.1 Category 1: Perceptions, Beliefs and Values Regarding the Practice of Bedside Milk Expression

When asked about the definition of BME, their opinions on the practice and the emotions it evoked, seven participants reported not practising BME and were unaware of its meaning. Two participants recognised that it involved expressing breast milk but did not specify that it was performed beside their baby in the NICU. Four participants reported expressing milk exclusively in the institution's milk bank extraction room, while only nine were able to accurately define the practice.

One notable aspect is that three mothers engaged in BME yet were unable to define the practice; two of them even conflated BME with human milk donation. Moreover, some narratives identified contact with the infant and the development of maternal–infant bonding as essential components of how they understood BME.

It's about expressing milk near the baby, in contact with them, looking at them to stimulate lactation in the NICU.(Calendula)

Regarding maternal perceptions of BME, some participants highlighted its importance for their baby, expressed concerns about milk production and described the experience as marked by insecurity and fear, as it was their first encounter with preterm birth. In addition, many participants described BME as a source of immunity and protection for the infant, referring to breast milk as the newborn's first ‘vaccine’.

Based on the participants' statements, the experience of performing BME and offering breast milk even in small quantities elicited feelings of happiness, bonding and a sense of participation in their infant's clinical improvement and developmental progress.

I thought I would only express milk at the milk bank because everything is new to me, the whole preterm birth issue. So, I was very happy to be able to participate in this way, already giving her the milk instantly. (Gazania)

The plurality of definitions and the associations with other practices, such as milk donation or standard milking, suggest that mothers anchor BME in previous family and social practices and references. At the same time, the attribution of emotional meanings such as love, protection and presence is objectified in concrete elements, such as the act of milking close to the baby while looking at them. These statements reflect how BME is socially represented not only as a purely technical activity, but also as a gesture of maternal participation and emotional connection.

### 3.2 Category 2: Incentives, Barriers and Perceived Improvements for the Implementation of Bedside Milk Expression

When asked about their motivations for practising BME, participants stated that their own baby was their greatest source of incentive. They also reported that the recognised importance of expressed milk for weight gain, clinical improvement, bonding and strengthening their child's immune system motivated them to continue the practice despite adversities.

My incentive is seeing my child well, regardless of anything. I make sacrifices, I come and do it […] because, as the nurse guided me, when you perform bedside milk expression for a preterm baby, in addition to applying it to their cheek to create antibodies from the colostrum, it provides more protection.(Iris)

Regarding perceived barriers, while some mothers reported positive and satisfactory experiences with BME in NICUs, many highlighted challenges such as the lack of designated spaces for mothers in the maternity ward, an insufficient number of chairs for comfort during milk expression, medical procedures coinciding with expression sessions and delays in offering expressed milk due to staff shortages, all of which hindered the practice.

The chairs are very limited, there are many mothers expressing milk, and sometimes we have to wait for another mother to finish before we can sit […] The main issue is the procedures; sometimes they perform medical procedures during feeding times. This should be better monitored. The babies end up waiting too long. Some mothers complain that when male nurses come in, they are more shy, but we know that they are there to work, so in my mind, that issue is already resolved. I don't really mind being afraid, you know, of showing it, I even turn away a little to avoid it, but I know they're there for work, right, they're not there to, you know, be mean, so it works out. (Gazania)

A frequently reported challenge was the feeling of shame and discomfort while practicing BME due to the presence of male professionals and fathers in the NICU. Another commonly mentioned barrier was overcrowding in the units.

A particularly noteworthy statement came from a participant who questioned the necessity and appropriateness of BME. Additionally, another participant expressed concerns that direct contact with the baby might pose a risk of bacterial transmission.

Suggested improvements included providing more information and encouragement to mothers, enhancing communication between healthcare professionals and patients, reducing overcrowding in NICUs and ensuring greater privacy for BME. As mentioned in relation to barriers, one suggestion for improvement would be to reduce the number of babies that healthcare staff have to care for.

Encourage mothers more, provide more information, because some don't practice it simply because they don't have enough knowledge about it. (Violet)

It is clear, therefore, that in this category, BME is socially represented as an act of maternal protection that faces structural and interpersonal challenges. The meanings attributed to it were anchored in emotionally charged ideas such as maternal love, the desire to avoid formula feeding and the perception of breast milk as essential to the baby's well‐being. On the other hand, the barriers were expressed through concrete symbols of discomfort.

### 3.3 Category 3: The Influence of the Healthcare Team and NICU Environment on the Practice of Bedside Milk Expression

A considerable number of participants described feeling welcomed in the NICU, particularly due to the supportiveness of the nursing staff. This welcoming atmosphere facilitated a sense of calm and confidence in engaging in BME. The presence, guidance and empathy of these professionals emerge as central elements in the mothers' lived experiences and appear to reduce the anxiety and insecurity often associated with the practice.

The support here has been wonderful. The NICU environment makes me a little embarrassed because there are a lot of people, but it is still welcoming because the nursing team makes me feel completely comfortable.(Orchid)

Conversely, the absence of proactive support from the healthcare team is perceived as a major obstacle. When mothers feel neglected or unsupported, they report a decline in motivation and confidence.

If a healthcare professional does not come to guide me, provide all the necessary information, and show me how to do it, I won't be able to. Right now, I feel somewhat unmotivated and afraid that I won't succeed.(Bromelia)

The mothers' experiences suggest that BME acquires meaning through the relationships, routines and emotional climate of the NICU. More than a merely physiological act, the practice becomes entwined with broader ideas of maternal care, presence and responsibility. The healthcare team, especially nursing professionals, plays an essential role in how mothers interpret and feel about this experience.

In this process, mothers seem to rely on familiar cultural references to navigate a complex and unfamiliar setting. Social images of the attentive nurse or the devoted mother do not remain abstract; they are subtly reinforced or redefined through daily interactions. The NICU, with its particular organisation and emotional tone, becomes part of how mothers make sense of their role and actions. What begins as a vulnerable and uncertain experience is, in some cases, gradually transformed into one of competence and legitimacy, depending on how the environment responds.

The meanings attributed to BME are fluid and shaped by personal emotions and the social environment. Supportive, empathetic settings foster confidence and connection, while lack of guidance or distant interactions generate doubt. These variations reflect how individual experiences are embedded in a broader, negotiated social context.

### 3.4 Anchoring and Objectification in the Construction of Social Representations

**FIGURE 1:**
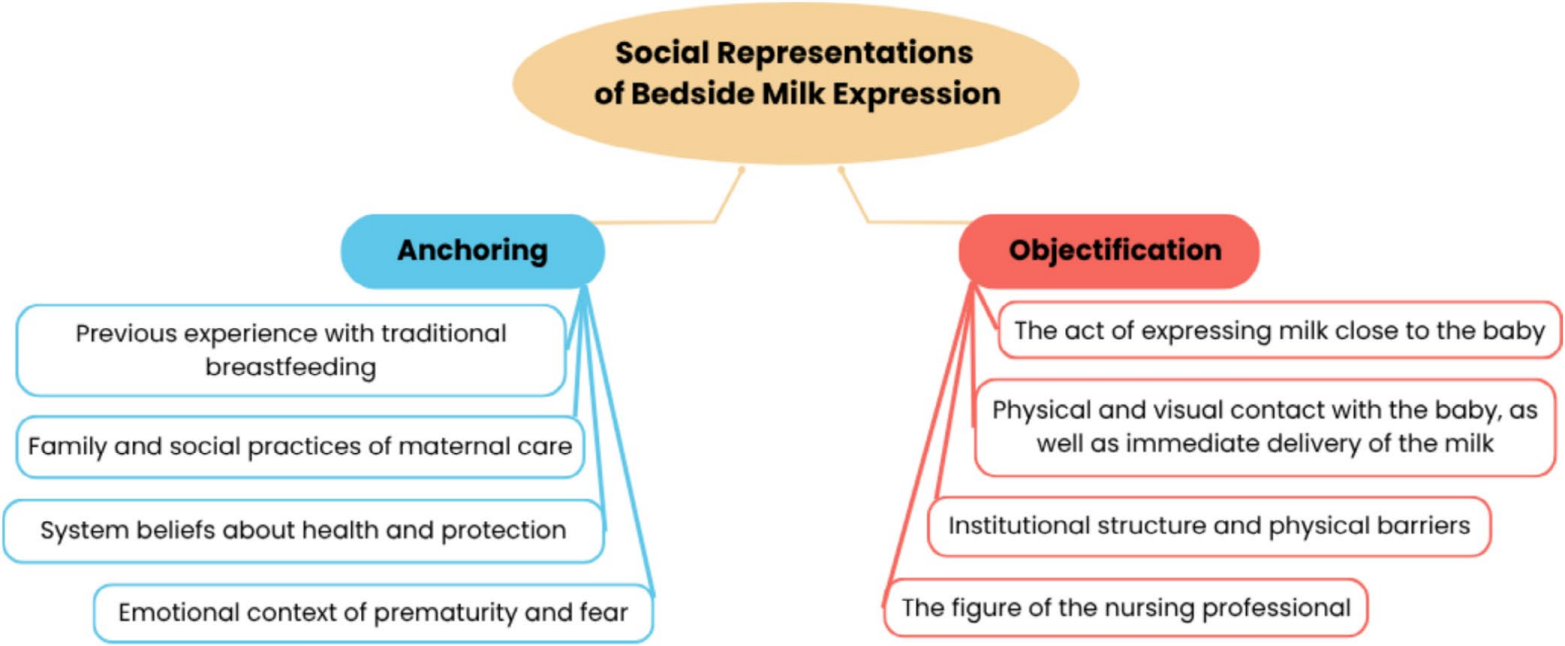
Anchoring and objectification processes in the social representations of bedside milk expression.

The findings demonstrate that mothers' social representations of BME are shaped by the processes of anchoring and objectification, which connect prior experiences, cultural references and symbolic meanings attributed to the practice. Figure 1 provides a visual synthesis of these processes, illustrating how mothers draw on familiar knowledge to interpret BME and transform abstract ideas into concrete images and practices within the NICU context.

In the anchoring process, BME is interpreted in the light of previous experiences with traditional breastfeeding, which is often confused with donating human milk, especially in the context of milk banks. This understanding is articulated with family and social practices of maternal care, in which milk takes on a symbolic place of efficacy and bonding. Anchoring also manifests itself in socially shared beliefs about health and protection, such as the conception of breast milk as the ‘first vaccine’, and in the intense emotions associated with prematurity, marked by fear, insecurity and the desire for the baby to recover.

These meanings are objectified in concrete and visible elements of the practice. The act of expressing milk close to the baby becomes a tangible form of care and presence, with the expressed milk being the materialisation of the maternal bond. Eye contact, touch, and the immediate delivery of milk transform feelings and intentions into observable gestures, reinforcing the perception of BME as an expression of affection and protection.

At the same time, objectification is expressed in the experience of concrete limits that interfere with the realisation of the practice, making the challenges faced by mothers during BME perceptible on a daily basis. In this context, the figure of the health professional, especially the nurse, appears as a concrete representation of the mediation between the mother's desire and the practical possibility of BME and can symbolise both a facilitator and an obstacle to carrying out this care.

## 4 Discussion

Three main categories were identified that permeate maternal social representations about the practice of BME: (1) perceptions, beliefs and values in relation to the practice of bedside milking; (2) perceptions, barriers and perceived improvements to the implementation of bedside milking and (3) the influence of the healthcare team and the NICU environment on the practice of bedside milking. These representations are shaped by their previous experiences, beliefs, culture and interactions with the healthcare team and hospital environment.

BME is characterised by the ambivalence between personal fulfilment through active participation in neonatal care and the physical, psychological and educational barriers encountered. Recognising these barriers and maternal social representations is crucial for strengthening breastfeeding among hospitalised preterm infants, as this practice fosters emotional bonding, supports the development of parenthood, and contributes to significant clinical improvements in newborns.

These maternal perceptions reveal the anchoring of BME in pre‐existing beliefs about breastfeeding and motherhood, particularly the idea of breast milk as an essential component of care aimed at re‐establishing their baby's health. In addition, objectification is seen when the abstract notion of feeling like an active agent in the improvement and care of your child is materialised through the act of milking milk at the bedside, even in the absence of breastfeeding directly.

In this context, the present study revealed a knowledge gap among participants regarding the concept of BME. A Hispanic study conducted with mothers of extremely preterm infants found that, shortly after birth, mothers realised their breastfeeding experience would not align with their initial expectations or with the representations shaped through conversations with mothers of full‐term infants. However, after receiving information about the importance of breast milk and the possibility of expressing it for their babies, most participants adopted the practice, including those who initially preferred formula feeding (Medina et al. 2019).

Additionally, a cross‐sectional study conducted in India with 400 participants indicated that 62% identified healthcare professionals as their primary source of breastfeeding information (Rajak et al. 2023). This finding suggests that the lack of guidance from healthcare professionals can negatively impact maternal and family knowledge, potentially affecting a mother's ability to breastfeed her preterm infant, thereby corroborating the findings of the present study.

Regarding maternal perceptions and emotions described in this study, it was observed that prematurity and NICU hospitalisation evoke feelings of insecurity among mothers. These emotional responses are essential for the construction of social representations. The evocation of these emotions, based on previous experiences and sociocultural expectations about motherhood, brings the symbolism of BME as a coping strategy and reaffirmation of maternal identity in the face of the current moment.

It is known that having a child admitted to a NICU is associated with severe parental anxiety and exhaustion, leading to feelings of despair, distress and disorientation (Jiang and Jiang 2022). A qualitative study involving parents of hospitalised infants highlighted that having a child in the NICU is a challenging and stressful experience, with separation from the newborn causing episodes of crying and sadness (Rihan et al. 2021).

Scientific literature strongly supports the concept of zero separation between mother and infant, as it prevents maternal–infant suffering, maintains milk production and strengthens exclusive breastfeeding (Brødsgaard et al. 2022; Bartick et al. 2021). It is recommended that mothers have unrestricted access to their infants and that institutions meet basic maternal needs, including food provision when possible (Ikonen et al. 2015).

A retrospective study with 865 preterm infants hospitalised in NICUs showed that families facing transportation difficulties had a 47% lower chance of breastfeeding at hospital discharge (Sankar et al. 2022). Furthermore, a qualitative study with mothers of extremely preterm infants revealed that the distance between home and hospital exacerbated mother‐infant separation, affected milk production and generated feelings of disconnection from the newborn (Medina et al. 2019).

The World Health Organization (WHO) and the United Nations Children's Fund (UNICEF) (2020) highlight that BME, combined with visual, auditory and tactile stimuli from mother‐infant contact, can enhance milk production. This underscores the importance of institutional strategies that support maternal presence with their babies.

Transportation, accommodation and food costs associated with maternal presence are recognised as barriers to breastfeeding and BME, negatively influencing maternal–infant contact, maternal self‐efficacy and the ability to cope with the trauma and stress caused by the infant's clinical condition (WHO and UNICEF 2020).

These structural and logistical challenges, already mentioned in the literature, are objectified in the statements of the participants in this study as tangible barriers to connecting with their babies and practising bedside milking. In this way, it can be understood that representations of BME are formed not only by emotional experience, but also by interaction with institutional conditions, which can be reinforcing or detrimental to the development of maternal capacity and autonomy to act as caregivers for their babies.

Additionally, offering formula to preterm infants in NICUs increases the risk of breastfeeding cessation after discharge (Jiang and Jiang 2022), a concern reflected in the statements of some participants in this study. This concern may be anchored in previous beliefs and experiences, built up in family and cultural contexts where breast milk is seen as a symbol of attachment, protection and maternal competence. Thus, the introduction of formula may have been interpreted as a threat to the continuity of breastfeeding and even to the experience of motherhood itself.

Other frequently reported barriers include the lack of privacy and overcrowding in NICUs, which can anchor the performance of BME in representations of stress, exposure and discomfort. These meanings can discourage the practice or associate it with vulnerability rather than security and support. The neonatal unit environment can significantly hinder breastfeeding and BME, as it is often noisy, excessively lit and lacks privacy, which may inhibit mother‐infant interaction (WHO and UNICEF 2020).

Studies indicate that mothers feel insecure and stressed by constant alarms, which negatively affect milk production, and report feelings of embarrassment in the presence of other parents and family members in the NICU (Medina et al. 2019; Brødsgaard et al. 2022). Strategies such as limiting foot traffic during BME and using privacy screens can promote maternal comfort.

Regarding mothers' concerns about medical procedures during BME and delays in offering expressed milk, it is understood that challenges such as limited professional support, separation from the infant and emotional distress can hinder NICU operations (Yang et al. 2019).

These concerns are objectified when these challenges materialise in experiences of waiting, frustration and interruptions to the flow of care. These elements come to represent for the mother not only operational failures, but barriers to building a bond and to the efficacy and validation of the very act of milking her milk at her baby's bedside.

A cross‐sectional study conducted in Kenya suggested a significant relationship between high levels of missed care and inadequate staffing, particularly in public health settings. This issue may impact the timing of infant feeding, among other factors (Gathara et al. 2020). Thus, inadequate staffing due to overcrowding and reduced professional availability can be obstacles to BME.

As reported by participants, BME fostered a sense of connection and belonging to their baby, contributing to their developmental and clinical progress. Similarly, in a Danish qualitative study, mothers of preterm infants described milk expression as a way to support their infants' survival and development, reinforcing their sense of uniqueness, as being able to breastfeed in some form validated their maternal identity (Brødsgaard et al. 2022).

Although motherhood encompasses much more than lactation, breastfeeding reinforces maternal identity as a provider and caregiver. The emotional bond with the infant influences the construction of social representations in various aspects, shaping maternal decisions and practices (Pombo‐de‐Barros and Arruda 2010).

Recognising milking as a form of bonding between mother and baby shows the objectification of maternal care, where a technical act gains symbolism by representing presence, protection and love. Furthermore, it can be inferred that this gesture is anchored to the socially constructed and perceived maternal identity, reinforcing the expression of milk at the bedside as a tangible manifestation of motherhood in the adverse context of prematurity and hospitalisation.

The importance of healthcare professionals, particularly nurses, in supporting and promoting information about breastfeeding and BME for mothers of preterm infants in NICUs is reiterated. This theme was recurrent in participants' accounts, with healthcare professionals recognised as key figures in overcoming existing barriers. Their actions serve as anchors that validate the practice or, when absent, contribute to evoking representations of incapacity or abandonment. When the healthcare team is attuned and responsive to the needs of the mother‐infant dyad, providing clear guidance and encouragement, mothers feel more supported and welcomed in the NICU environment (Flacking et al. 2021).

Healthcare professionals in NICUs play a critical role in breastfeeding and BME practices for preterm infants, offering direct support and health education. Professional support helps mothers navigate NICU‐related challenges, fostering greater confidence and autonomy in breastfeeding, even after hospital discharge (Tortorella et al. 2024). A retrospective study indicated that the presence of lactation specialists in NICUs significantly increased human milk usage among extremely and late preterm infants (Sankar et al. 2022).

Nursing support facilitates BME by reshaping social representations of its complexity and creating a more welcoming, breastfeeding‐friendly environment. This relationship reflects the interactive nature of SRT, in which representations are constructed collectively through communication and shared experience. The nurses' technical guidance and emotional support help mothers to feel confident and able to express milk, overcoming psychological and structural barriers, as well as helping them to see BME not as an obligation, but as an important part of their maternal care.

It is noteworthy that breastfeeding is socially perceived as a natural and instinctive female activity. Mothers are often the primary caregivers, having the most contact with hospitalised infants. Despite the challenges of maintaining lactation, these difficulties do not diminish their motherhood. Healthcare professionals encouraging BME should understand how mothers process this experience and foster autonomy in making informed decisions for themselves and their babies (Tortorella et al. 2024).

Understanding these aspects and representations is crucial for developing care plans that address the real needs of this population, taking into account the knowledge and social representations that influence maternal experiences. In this study, the SRT structure reinforces that breastfeeding and bedside milking are not purely technical actions, but socially constructed experiences full of personal and cultural meanings. This reflects the complexity of care, marked by subjectivity and the specific challenges inherent in breastfeeding premature babies.

Therefore, further training for healthcare professionals on this topic is essential, as each maternal experience is unique and requires individualised care. Additionally, studies like this prompt reflection on the type of care provided and the NICU environment, as intensive care settings often prioritise technical approaches. This underscores the need for more humanised care and enhanced maternal support within neonatal units.

## 5 Limitations

Finally, a limitation of this study is that it was conducted with a single group of mothers of preterm infants in a NICU at a referral maternity hospital in Fortaleza, Brazil. Therefore, the social representations observed and discussed cannot be generalised. However, it is believed that these findings may resonate with the experiences of other mothers of preterm infants in NICUs or similar contexts, contributing to further research and improvements in maternal care and NICU environments.

## 6 Conclusion

The social representations of bedside milking among mothers of premature newborns admitted to the NICUs are permeated by affective, symbolic and social meanings. The practice was often anchored in previous breastfeeding experiences and in the values attributed to breast milk as an expression of maternal bonding, protection and presence. Objectification was manifested in the realisation of milking as an act of care and exercise of motherhood in an environment sometimes marked by technification, where closeness is not always preserved in care interactions.

These representations, built from the articulation of emotional experiences, previous knowledge and interactions with health professionals, show that milking goes beyond a technical practice; it acquires a powerful subjective dimension that needs to be recognised by multi‐professional teams. Considering these social constructions can favour more empathetic, welcoming and effective approaches, guided by an understanding of the meanings that women attribute to their care experience.

Analysis anchored in SRT thus contributes to broadening the understanding of BME in the NICUs as a socially constructed phenomenon, allowing clinical policies and practices to be more sensitive to maternal subjectivities. Strengthening support for BME, while respecting the meanings it carries for mothers, can promote the continuity of breastfeeding, the mother‐baby bond and the humanisation of neonatal care.

Future research should investigate the social representations of BME in different contexts and regions. Additionally, it is necessary to assess the impact of educational interventions, professional training and structural improvements to strengthen breastfeeding and promote more humanised care practices.

## Author Contributions

Ana Karen de Sousa Alves: conceptualisation, methodology, investigation, data curation, formal analysis, visualisation, writing – original draft, writing review and editing. Ana Paula Melo Façanha: conceptualisation, resources, supervision, project administration, validation, writing – review and editing. Elaine Meireles Castro: supervision, validation, formal analysis, writing – review and editing. Keline Soraya Santana Nobre: supervision, validation, writing – review and editing. Flávia Vasconcelos Teixeira: supervision, validation, writing – review and editing. Maria Milena Farias de Souza Castro: supervision, validation, writing – review and editing. José Mateus Pires: supervision, validation, writing – review and editing. Victórya Suéllen Maciel Abreu: supervision, validation, writing – review and editing. Priscila de Souza Aquino: supervision, validation, writing – review and editing.

## Ethics Statement

The Research Ethics Committee of the Assis Chateaubriand Teaching Maternity Hospital, Fortaleza, Ceará, Brazil approved this study (approval number 6.918.137, 28 June 2024). The study complied with the bioethical principles established by Resolution No. 466/12 of the National Health Council, Brazil.

## Consent

All participants signed the Free and Informed Consent Form prior to data collection and confidentiality and anonymity were ensured by assigning flower names to participants.

## Conflicts of Interest

The authors declare no conflicts of interest.

## Data Availability

The data that support the findings of this study are available from the corresponding author upon reasonable request.
